# Overexpression of Glutathione S-Transferases in Human Diseases: Drug Targets and Therapeutic Implications

**DOI:** 10.3390/antiox12111970

**Published:** 2023-11-06

**Authors:** Ning Lv, Chunyan Huang, Haoyan Huang, Zhiqiang Dong, Xijing Chen, Chengcan Lu, Yongjie Zhang

**Affiliations:** 1 Clinical Pharmacology Research Center, School of Basic Medicine and Clinical Pharmacy, China Pharmaceutical University, Nanjing 211198, China; 3222091980@stu.cpu.edu.cn (N.L.); 3121090296@stu.cpu.edu.cn (H.H.);; 2 Department of Pharmacy, The Affiliated Jiangning Hospital of Nanjing Medical University, Nanjing 211100, China; zqdongjnyy@njmu.edu.cn; 3 Jiangning Clinical Medical College, Jiangsu University, Nanjing 211100, China

**Keywords:** glutathione S-transferases, overexpression, chemoresistance, neurodegenerative disease, pulmonary fibrosis, GST inhibitors

## Abstract

Glutathione S-transferases (GSTs) are a major class of phase II metabolic enzymes. Besides their essential role in detoxification, GSTs also exert diverse biological activities in the occurrence and development of various diseases. In the past few decades, much research interest has been paid to exploring the mechanisms of GST overexpression in tumor drug resistance. Correspondingly, many GST inhibitors have been developed and applied, solely or in combination with chemotherapeutic drugs, for the treatment of multi-drug resistant tumors. Moreover, novel roles of GSTs in other diseases, such as pulmonary fibrosis and neurodegenerative diseases, have been recognized in recent years, although the exact regulatory mechanisms remain to be elucidated. This review, firstly summarizes the roles of GSTs and their overexpression in the above-mentioned diseases with emphasis on the modulation of cell signaling pathways and protein functions. Secondly, specific GST inhibitors currently in pre-clinical development and in clinical stages are inventoried. Lastly, applications of GST inhibitors in targeting cell signaling pathways and intracellular biological processes are discussed, and the potential for disease treatment is prospected. Taken together, this review is expected to provide new insights into the interconnection between GST overexpression and human diseases, which may assist future drug discovery targeting GSTs.

## 1. Introduction

Glutathione S-transferases (GSTs) were first isolated from cytoplasm in rat liver tissue in the 1960s and have been of continuous research interest ever since [1]. Mammalian GSTs are a large family that can be further divided into three classes, namely cytosolic GSTs, mitochondrial GSTs, and microsomal GSTs, according to their cellular localizations [2]. Among them, cytosolic GSTs are probably the most well-studied GSTs and are widely expressed in various types of cells [3]. Therefore, this review mainly focuses on cytosolic GSTs, their different biological activities, and their roles in human diseases.

Cytosolic GSTs exist as homodimers or heterodimers in the cytoplasm of cells, with a subunit length between 200 and 250 amino acids and a molecular weight between 23 and 28 kDa [4,5,6]. Cytosolic GSTs are classified into seven classes based on the similarity of amino acid sequences and structural features: Alpha (α), Sigma (σ), Mu (μ), Pi (π), Omega (ω), Theta (θ), and Zeta (ζ) [7,8,9]. In mammals, the sequence identity of cytosolic GST isozymes in the same class is >40%, and the sequence identity of isozymes between classes is <25% [10]. Each isoform is encoded by a unique gene, and the coding genes are in different chromosomal locations. Cytosolic GSTs are extensively expressed in human tissues (Table 1). Cytosolic GSTs have a variety of biological functions: (1) catalysis of conjugation reactions of reduced glutathione (GSH) to electrophilic substances (including drugs), electrophilic drug metabolites, and endogenous electrophiles [5]; (2) catalysis of reduction in organic hydroperoxides; (3) regulation of various cellular signaling pathways, such as the mitogen-activated protein (MAP) kinase pathway via the inhibition of c-Jun N-terminal kinase 1 (JNK1) and apoptosis signal-regulating kinase 1 (ASK1) [11]; (4) post-translational modification of various proteins by S-glutathionylation or de-glutathiolation [12]; and (5) contribution to multidrug resistance to chemotherapeutic drugs and protection of cancer cells against apoptosis [13].

A growing number of studies have associated the diverse biological activities of GSTs with a variety of diseases. GSTs have been shown to be overexpressed in many tumor tissues. This high expression of GSTs mediates cellular resistance to antitumor drugs via distinct mechanisms, which mainly involve metabolic detoxification, regulation of the MAPK signaling pathway, DNA repair, autophagy, and glycolytic processes. In addition, a recent study showed that a high expression of GSTs in lung fibroblasts promotes the progression of pulmonary fibrosis by catalyzing protein S-glutathionylation in lung fibroblast [22]. In addition, the complex role of GSTs in the pathogenesis of neurodegenerative diseases is also attracting increasing research interest [23,24]. In this review, we focus on the consequences of overexpression of GSTs in a selection of human diseases, including tumor resistance, pulmonary fibrosis, and neurodegenerative diseases. In addition, the development of GST inhibitors to suppress overexpressed GSTs as possible therapeutic implications was introduced.

## 2. Structure

**Figure 1:**
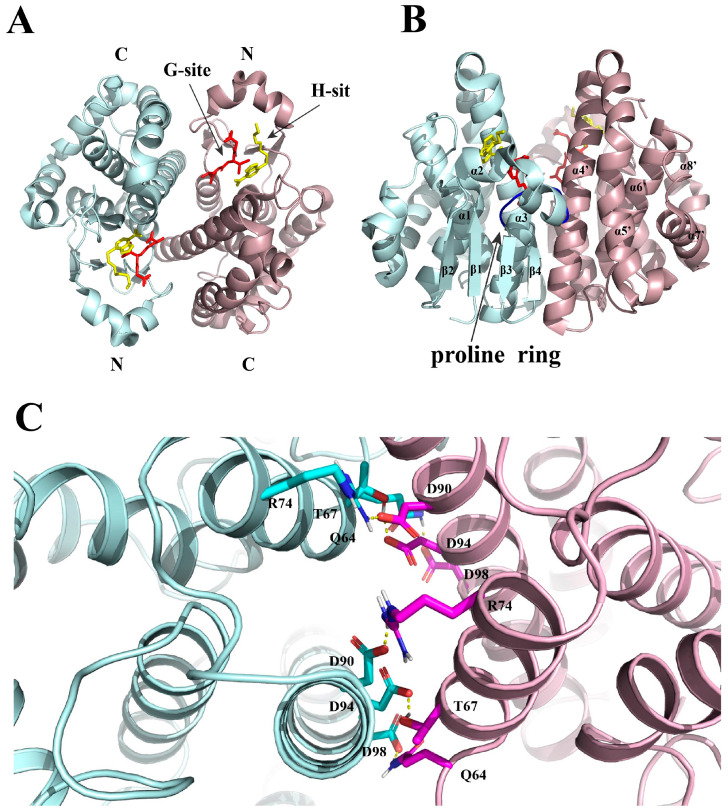
Structural diagram of human GSTP1 (pdb code 3GUS). (**A**) The two subunits are colored blue and pink, respectively. The G site is occupied by a GSH (red rod structure) molecule, while the H site is occupied by a NBDHEX (yellow rod structure) molecule. (**B**) The thioredoxin-like folded structure consists of four β-folds and three α-helices in the order β1-α1-β2-α2-β3-β4-α3, with α2 and β3 connected by a proline ring. (**C**) Interactions of amino acids at the interface of two subunits. The figure is created using PYMOL version 2.6.0a0.

As mentioned above, GST active enzymes are composed of homo- or heterodimeric forms. Each subunit contains two functional domains: the N-terminal functional domain and the C-terminal functional domain. The two functional domains correspond to two active sites, namely the G-site and H-site, respectively (Figure 1A) [25]. The N-terminal functional domain is made up of amino acids 1–82, with a thioredoxin-like folded structure consisting of four β-folds and three α-helices (βαβαββα) (Figure 1B) [26]. Starting from the N-terminal and ending at the C-terminal, the order is β1-α1-β2-α2-β3-β4-α3, where β1β2β4 are parallel in the same direction, and with β3 in the opposite direction [27]. Helix α2 and fold β3 are connected through a proline ring consisting of cis-proline residues (Figure 1B). This ring is not directly involved in the catalytic function of GSTs but forms hydrogen bonding interactions with the backbone amine group of the cysteine-residue of GSH and plays an important role in maintaining the protein in a catalytically active structure [28,29]. The N-terminal functional domain is the binding site for GSH and is therefore referred to as the G site. This site has a strong specificity for GSH and is therefore a very conserved pocket [30]. N-terminal amino acid residues important for GSH activation include serine (GSTT), tyrosine (GST A, M, S, and P), or cysteine (GSTO and Z) [16]. The hydroxyl group of the Tyr/Ser residue forms a hydrogen bond with the sulfhydryl group of GSH to promote the formation and stabilization of the thiolate anion. In contrast, the Cys residue of GSTO and GSTZ forms a mixed disulfide with GSH, not a thiolate anion [27]. It is noteworthy that GSTs containing different amino acid residues have different catalytic activities. For example, most GSTs containing serine or tyrosine residues (GSTT/GSTA/GSTM/GST/GSTP) exhibit glutathione transferase activity, while GSTs with catalytic cysteine residues (GSTO/GSTZ) exhibited glutathione lyase activity [25].

The C-terminal functional domain consists of amino acids 90–217 and is an all-α helical structural domain [31]. It is the binding site for hydrophobic compounds and is therefore designated as the H site [32]. The H site is not conserved and can non-specifically bind to a large variety of hydrophobic substrates with varying affinities, such as heme, bilirubin, dexamethasone, and polycyclic aromatic hydrocarbons [33].

These two sites jointly form a complete subunit from amino acid residues at positions 83–89, which is recognized as the active catalytic site of GSTs. The two subunits interact via the contact between the N-terminal structural domain of one subunit and the C-terminal structural domain of the other subunit, and this interaction is mainly electrostatic and hydrophobic (Figure 1C) [26]. The catalytic mechanism of two sites involved in GSTs is as follows: (1) GSH binds to the G site to form the strong nucleophilic thiolate anion, (2) the electrophilic substrate bound to the H site reacts with the thiolate anion of GSH to form the GSH conjugate, which will be released via the C-terminus [34].

## 3. Physiological Function

### 3.1. Detoxification

GST are important phase II detoxification enzymes involved in the detoxification of a variety of exogenous and endogenous substances. The general formula of a GST-catalyzed substitution reaction is presented as GSH + R-X → R-SG + H-X, where the X represents a leaving group, for example, halogen (Cl, Br, I) or sulfate groups. In addition, GSTs can catalyze additional reactions, as in the case of α, β-unsaturated aldehydes, epoxides, quinones, and quinoneimines.

**Figure 2:**
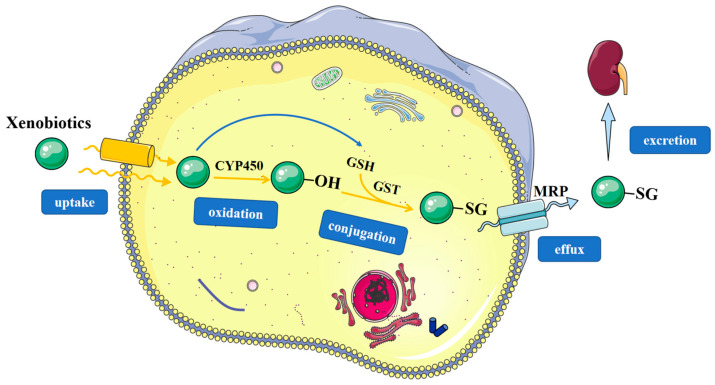
The involvement of GSTs in detoxification process of xenobiotics. The xenobiotics can be divided into lipophilic compounds and hydrophilic compounds. Lipophilic compounds are metabolized by the phase I metabolic enzyme CYP450s, resulting in an increased polarity. Polar metabolic intermediates or hydrophilic compounds are metabolized by phase II metabolic enzymes like GSTs with formation of highly water-soluble conjugate R-SG. R-SG is then excreted from the cell by multidrug resistance proteins (MRPs) and finally processed into mercapturic acid conjugates that are excreted in the urine.

The hydrophilic GSH-conjugate (R-SG) formed intracellularly is excreted from the cell by the multidrug resistance-associated protein MRP and then processed subsequently by gamma-glutamyltransferases, dipeptidases and N-acetyltransferases to an N-acetylcysteine conjugate, also known as mercapturic acid, which will be excreted in the kidney to urine [35]. Various electrophilic compounds are known to cause intracellular damage by covalently binding to DNA and proteins. Thus, the GST-catalyzed GSH conjugation is protective of these biomolecules inside the cell and therefore is considered as a detoxification process (Figure 2) [9,36]. The major types of GST-catalyzed reactions include epoxide ring opening, nucleophilic aromatic substitution reactions, Michael addition of α, β-unsaturated aldehydes and ketones, isomerization, and peroxidase reactions [5,37].

**Figure 3:**
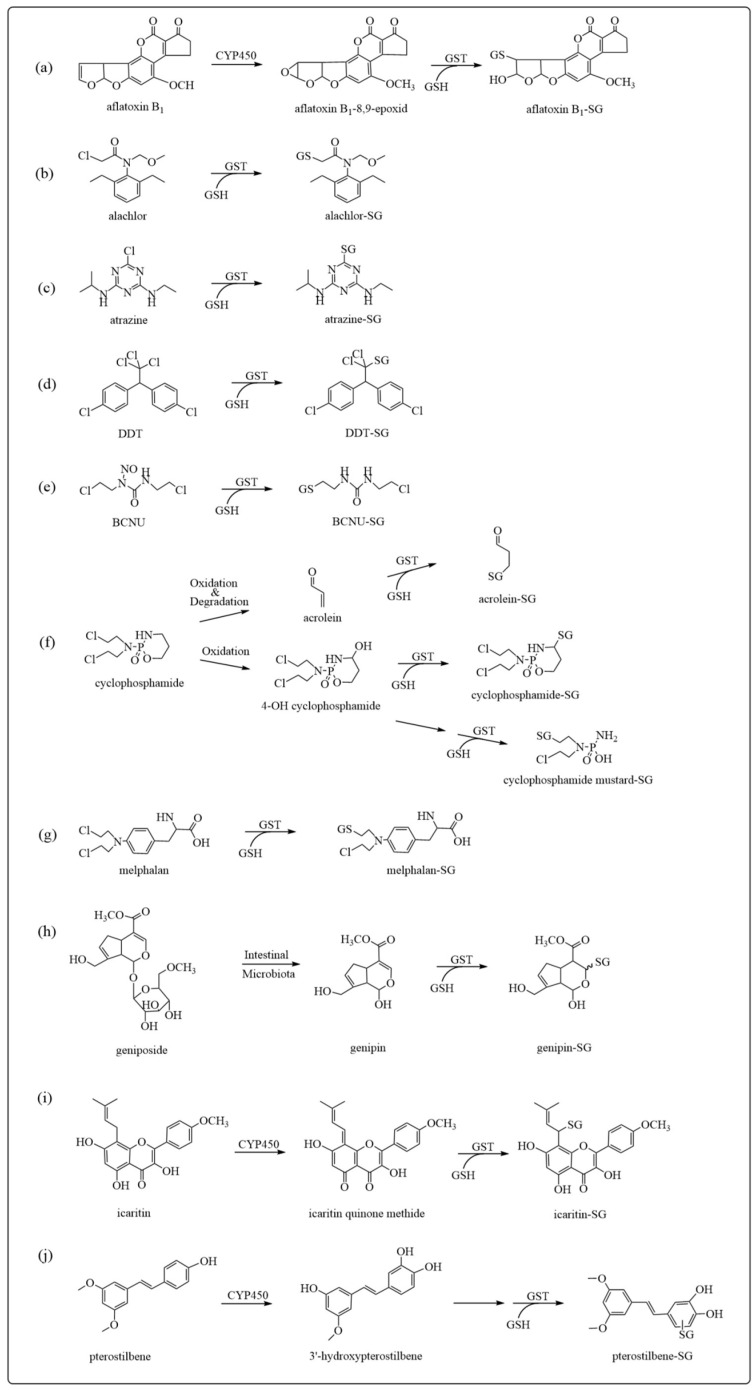
Examples of exogenous compounds as GST substrates: (**a**) aflatoxin B1; (**b**) alachlor; (**c**) atrazine; (**d**) DDT; (**e**) BCNU; (**f**) cyclophosphamide; (**g**) melphalan; (**h**) geniposide; (**i**) icaritin; (**j**) pterostilbene.

The substrate selectivity of GSTs is very broad and includes both exogenous and endogenous compounds, as illustrated by the examples shown in Figure 3. For example, GSTs inactivate the 8,9-epoxide formed by the CYP450-catalyzed oxidation of the environmental toxicant/carcinogen aflatoxin B1, Figure 3a. The pesticides alachlor atrazine and dichlorodiphenyltrichloroethane (DDT) are metabolized directly by GSTs by chloro-substitution reactions, which in the case of DDT is followed by spontaneous deglutathionylation leading to DDE (Figure 3b–d) [38,39]. The metabolism of anticancer drugs by GSTs is a process that leads to loss of drug efficacy, as in the case of 1,3-di-(2-chloroethyl)-1-nitrosourea (BCNU), cyclophosphamide and melphalan (Figure 3e–g) [40]. Also, certain components of medicinal herbs are known to be metabolized to reactive metabolites that have been associated with toxic reactions. GST-mediated detoxification reactions have been recently reported for reactive metabolites derived from geniposide, icaritin, and pterostilbene (Figure 3h–j) [41,42,43].

**Figure 4:**
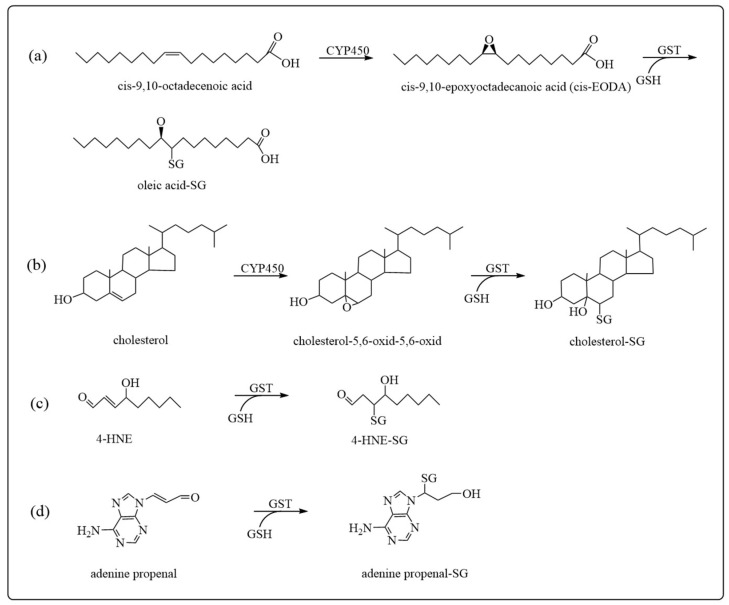
Examples of oxidative stress products as GST substrates: (**a**) oleic acid; (**b**) cholesterol; (**c**) 4-HNE; (**d**) adenine propenal.

Some endogenous compounds, such as oleic acid, arachidonic acid, and cholesterol, are metabolized by the cytochrome CYP450 enzyme to reactive epoxides (Figure 4a,b). GSTs have been shown to catalyze the inactivation of these epoxides by GSH conjugation [44,45,46]. Another source of endogenous electrophiles is the process of lipid peroxidation and oxidation of DNA bases. These processes can be initiated by reactive oxygen species (ROS) formed by ionizing radiation, aerobic respiration, or inflammation. ROS, which include superoxide anion radicals, hydrogen peroxide, and hydroxyl radicals, can interact with membrane lipids and DNA to produce toxic metabolites containing lipid peroxides, carbonyls, and epoxides [37]. Decomposition of lipid peroxides can lead to protein-reactive metabolites, e.g., 4-hydroxynonenal, whereas the oxidation of DNA bases can lead to reactive adenine propenal (Figure 4c,d) [38,47]. Studies have shown that electrophilic substances produced by oxidative stress are associated with a variety of diseases, such as cancer, Alzheimer’s disease, Parkinson’s disease, schizophrenia, diabetes, atherosclerosis, and aging-related diseases [48,49,50,51,52,53,54]. GSTs play an important role in the inactivation of these endogenous electrophiles, which implies that a deficiency of GSTs may increase the risk for these diseases.

### 3.2. Cellular Signaling Regulation

#### 3.2.1. JNK Signaling Pathway

**Figure 5:**
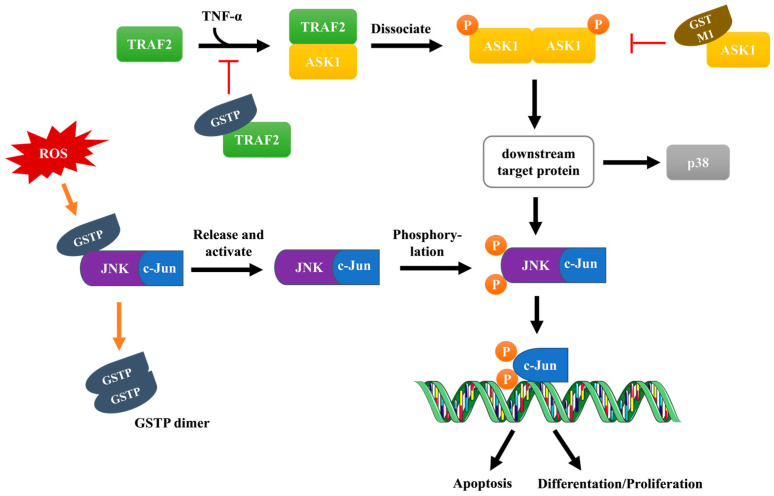
Regulatory roles of GSTP1 in the MAPK pathway. (1) GSTP1-JNK interaction: Under conditions of oxidative stress, GSTP1 dissociates from the heterotrimeric complex formed with JNK/c-Jun, releases and activates the JNK/c-Jun complex and forms a GSTP1 dimer. After that, JNK and c-Jun are phosphorylated and activated successively, and c-Jun is involved in transcription and regulates cell growth (apoptosis, proliferation, or differentiation). (2) GSTP1-TRAF2 interaction: When cells are stimulated by TNF-α, intracellular ROS is generated and ASK1 is activated and binds to TRAF2, ultimately activating JNK and p38. GSTP1 can form a complex with TRAF2 to prevent this process, while GSTM1 plays a regulatory role by directly acting on ASK1.

Apart from the catalytic functions, GST isoenzymes are also involved in the mitogen-activated protein kinase (MAPK) pathway, a cell survival and death signal transduction pathway, in a non-catalytic manner by direct protein–protein interaction [18]. C-Jun N-terminal kinase (JNK), a member of the MAPK superfamily, plays an important role in the regulation of cell proliferation, apoptosis, inflammatory response, cancer, fibrosis, and other pathophysiological processes [55]. Many studies have shown that GSTP1 interacts with JNK and inhibits its activity, thereby suppressing the activation of downstream targets [56,57,58]. In the non-stressed cellular status, JNK activity is strongly suppressed by GSTP1 binding, Figure 5. It has been shown that the activity of JNK is negatively correlated with the expression level of GSTP1, confirming the negative regulatory role of GSTP1 [58]. Once cells are stimulated by cytokines, radiative, oxidative, or other types of stress, GSTP1 dissociates from JNK, and the activated JNK mediates phosphorylation of c-Jun, a member of the activated protein-1 (AP-1) transcription complex. Phosphorylated c-Jun, in turn, regulates biological processes such as cell proliferation and apoptosis [59]. It has been confirmed that GSTP1 is highly expressed in a variety of cancer cells [60,61,62,63], which is considered an essential mechanism of multidrug resistance to chemotherapeutics because suppression of JNK leads to the prevention of apoptosis. GSTA1 and GSTM1 can also form protein complexes with JNK in a mechanism similar to GSTP1, but their inhibitory activity against JNK is rather weak [2,36].

#### 3.2.2. ASK1 Signaling Pathway

GSTP1 also regulates upstream signaling of the tumor necrosis factor-α (TNFα) activated MAPK pathway. In human cervical cancer Hela cells, overexpression of GSTP1 was shown to not only inhibit the activation of JNK, as described above but also activation of p38 by suppression of the tumor necrosis factor receptor-related factor 2 (TRAF2), thereby playing a key regulatory role in TNF-α-induced MAPK signal transduction [11,64]. GSTP1 forms a complex with TRAF2 and thereby prevents the formation of TNF-α-induced TRAF2-ASK1 complex and therefore suppresses the activation of apoptosis signal-regulated kinase 1 (ASK1) [65]. Since ASK1 is a MAPK kinase that activates the JNK and p38 pathways, GSTP1-mediated indirect inhibition of ASK1 results in cells being spared from apoptosis (Figure 5) [66]. Interestingly, GSTM1 can also mediate similar inhibitory effects, but unlike GSTP1, GSTM1 is able to directly bind and inhibit the activity of ASK1 [67]. In addition, GSTM3 has been reported to interact with TRAF6 in cervical cancer tumors similar to GSTP1-TRAF2 [68].

#### 3.2.3. Other Signaling Pathways

Dowling et al. [69] showed that the activation of AMPK can inhibit the mammalian target of rapamycin (mTOR), resulting in the blockade of the synthesis of downstream proteins. Therefore, activating AMPK to inhibit mTOR is a potential anti-cancer strategy. AMPK activity has been shown to be suppressed by GSTP1. It was shown that the inactivation of GSTP1 in triple-negative breast cancer (TNBC) resulted in decreased cell survival and tumorigenesis of TNBC cells, which is related to decreased suppression of AMPK activation by GSTP1 [70]. Other GST isozymes, such as GSTA1, negatively regulate the mTOR signaling pathway [71]. GSTO1 is able to regulate the activation of protein kinase B and MAPK1/2 [72]. Studies in human hepatoma cells have shown that GSTP1 is able to bind to signal transduction and transcriptional activator 3 (STAT3) to form the GSTP1-STAT3 complex, inhibiting epidermal growth factor (EGF)-mediated phosphorylation of STAT3 tyrosine and preventing its transcriptional activity, thereby reducing cell proliferation and halting the cell cycle [73].

### 3.3. Protein S-Glutathionylation

Post-translational modifications (PTMs) of proteins are important processes that regulate the activity of proteins by adding chemical groups to one or more critical amino acid residues [74]. Common forms of PTMs include phosphorylation, acetylation, ubiquitination, methylation, glycosylation, SUMOylation, carbonylation, etc. [13]. Among them, S-glutathionylation is a process of forming mixed disulfide bonds between protein cysteine residues and the cysteine residue of GSH [75]. This process is reversible and is also thought to be a protective mechanism against irreversible modification of the cysteine sulfhydryl groups of target proteins [76,77]. Correspondingly, deglutathionylation of proteins refers to the release of the GSH group from the protein cysteine residue, which reduces protein to its native status [78,79]. S-glutathionylation of proteins is normally induced by endogenous oxidative stress or nitrosative stress (RNS) but can also be the result of exposure to exogenous oxidants [80].

S-glutathionylation of proteins and its reverse reaction deglutathionylation are both spontaneous and enzymatic. The major enzymes catalyzing forward S-glutathionylation reactions include GSTP1, GSTA4, and GSTO1, among which the role of GSTP1 was most recognized up to date. In the meanwhile, GSTO1, Grx, and Trx are major enzymes catalyzing deglutathionylation reactions [81,82,83]. Experiments showed that GSTP1 knockout mice had substantially lower overall protein glutathionylation levels than wild-type mice [84]. It has been reported that many proteins involved in various intracellular processes are prone to glutathionylation. Cellular processes affected by protein glutathionylation include protein folding and stability, nitric oxide regulation, and the activity of cysteinases involved in redox homeostasis. Glutathionylated proteins identified include cytoskeletal proteins, transcription factors, signaling proteins, ras and heat shock proteins, ion channels, calcium pumps and binding proteins, and glycolysis enzymes [80]. Proteins regulated by glutathionylation have been identified using proteomic and bioinformatic approaches. Several comprehensive reviews have been published which described the types of proteins susceptible to glutathionylation and the effects of post-translational modifications on their functions in detail [3,80,85]. For example, GSTP1 catalyzes the glutathionylation of oxidized peroxiredoxin VI (Prx VI), thereby restoring its peroxidase activity [86,87]. GSTP1 can also catalyze the glutathionylation of pyruvate kinase M2 (PKM2) and reprogram the classic glycolysis activity of PKM2. Increased GST expression and glutathionylation level of p53 block its ability to recognize common DNA sequences, thus promoting tumorigenesis [3,88,89]. The consequences of protein glutathionylation are complicated by modulating various physiological processes such as protein folding, cytoskeleton remodeling, signal transduction, inflammation, calcium homeostasis, and regulation of metabolic pathways [90]. Excessive protein glutathionylation was shown to be associated with various diseases, including tumorigenesis [91], antitumor drug resistance [92], cardiovascular diseases [93], and neurodegenerative diseases [94,95], which is to be discussed in the following sections within this review.

## 4. Roles of GST Overexpression in Human Diseases

### 4.1. GSTs and Tumor Multidrug Resistance

Chemotherapy is one of the most common and effective treatments for cancers. However, tumor cells are known to often develop multidrug resistance (MDR) during chemotherapy, which is the main reason for therapeutic failure [7]. The American Cancer Society estimates that more than 90% of cancer deaths are associated with MDR. MDR is defined as loss of sensitivity to antineoplastic drugs with distinct structures and different molecular targets. Many mechanisms have been proposed to explain MDR, such as the decrease in intracellular drug concentrations due to efflux pump induction, the mutation of drug targets, the upregulated metabolic detoxification, and the enhanced DNA damage repair function [63]. The mechanism of drug resistance may involve a variety of proteins. One type of MDR is based on overexpression of efflux pumps at the plasma membrane, such as P-gp, MRP1, and BCRP, resulting in strongly reduced intracellular drug concentrations [12]. Overexpression of P-gp is considered to play an important role in MDR and is the main reason for the failure of chemotherapy [96]. The other type of MDR is based on overexpression of GSTs, which can result in direct detoxification of chemotherapeutics and/or inhibition of the MAPK signaling pathway [12]. GSTs can cooperate with efflux transporters and multidrug resistance proteins to protect tumor cells from the cytotoxicity of anticancer drugs [97].

The role of GSTs, especially GSTP1, in the development of cancer has attracted attention in recent years. A study showed that the expression of GST isozymes is upregulated in 60 human tumor cell lines, both at mRNA and protein levels. GSTP1 was shown to be the most abundant isozyme in all of these cell lines [18,98]. Overexpression of GSTP1 has been reported to involve cancer cell resistance to chemotherapeutics, such as resistance of ovarian cancer cells against carboplatin and cisplatin, adriamycin-resistance of breast cancer cells and prostate cancer cells, resistance of gastric cancer cells against fluorouracil (5-FU) and cisplatin, and resistance of neurogliomas against cisplatin and irinotecan [99,100,101,102,103]. The roles of other GST isoforms, including GSTA, GSTM, GSTO, and GSTT, in MDR have also been investigated. For instance, it is demonstrated that GSTA played an essential role in the detoxification of chlorambucil via catalyzing the GSH conjugation reaction of this alkylating reagent [104]. Table 2 lists the involvement of different GST isoforms in drug-resistant chemotherapies and related antineoplastic drugs. Several GST isoforms have been shown to play essential roles in tumorigenesis and metastasis. For example, in breast cancer cells, chemotherapy-induced GSTO1 expression leads to chemotherapy resistance and promotes metastasis. The GSTO1 inhibitor S2E increased the rate of apoptosis by tamoxifen in MDA-MB-231 cells [105]. Wang et al. [106] showed that overexpressed GSTA1 not only promotes the proliferation of lung cancer cells but also stimulates metastasis of lung cancer cells by promoting epithelial–mesenchymal transition (EMT).

#### 4.1.1. Nuclear Localization of GSTP1

In addition to the high expression of GSTP1 in tumor tissues, the subcellular localization in tumor cells has been associated with oncogenic effects [2]. In normal cells, GSTP1 is mainly expressed in the cytoplasm, whereas it is found that in oral squamous cell carcinoma, GSTP1 is mainly located in the nucleus [118]. Nuclear GSTP1-negative cells have previously been shown to be more sensitive to cytotoxic drugs than nuclear GSTP1-positive cells, suggesting that the nuclear localization of GSTP1 is associated with drug resistance [119]. GSTP1 has been reported to be expressed in the nuclei of glioma cells and uterine cancer cells, and its nuclear localization showed a negative correlation with patient survival [120,121]. Rolland et al. [110] elaborated on the effect of GSTP1 nuclear translocation inhibitors on the chemotherapy sensitivity in mantle cell lymphoma (MCL) cells. It was shown that inhibition of GSTP1 nuclear translocation with agaricus bisporus lectin (ABL) was able to increase the sensitivity of MCL to doxorubicin (DOX), cisplatin (CDDP), cytarabine (Ara-C), gemcitabine (GEM), and bortezomib [110]. It is proposed that the nuclear localization of GSTP1 is chemotherapy-induced and contributes to the drug resistance of cancer cells.

#### 4.1.2. Effects of GSTs on Glycolysis

Glycolysis is one of the most important processes in cellular energy metabolism, converting glucose in cells to provide the energy needed for life activities. In tumor tissues, aerobic glycolysis with abnormal release of adenosine triphosphate (ATP) and lactate, which is known as the Warburg effect, fulfills the requirement of rapid tumor growth [115,122,123]. Lactate dehydrogenase A (LDHA) is known to be relevant to angiogenesis, proliferation, immune evasion, and metastasis during tumorigenesis [124]. Furthermore, altered glycolytic metabolism in tumor cells is highly correlated with the prognosis of tumor patients and therefore can be used as a target for cancer therapy [125]. A previous study showed that GSTM3 was highly expressed in TMZ-resistant T98G cells and affects glycolysis [115]. The activity of LDHA and the glycolytic end product L-lactate level were significantly reduced in T98G cells along with GSTM3 gene suppression, which implied that GSTM3 downregulation might prevent cell invasion. Interestingly, Wang et al. [126] found that GSTM3-silenced pancreatic cancer (PC) cells exhibited increased levels of glycolysis, whereas the overexpression of GSTM3 showed a decrease in glycolysis. This suggested that GSTM3 may provide a potential therapeutic strategy for PC treatment.

#### 4.1.3. Effects of GSTs on DNA Repair

DNA repair is a cellular response to DNA damage that will restore the DNA structure to its original form. However, it sometimes does not completely eliminate the DNA damage but only enables the cell to tolerate the DNA damage and continue to survive. Cancer cells use residual DNA repair capacity to repair the damage caused by DNA replication stress and genotoxic antitumor drugs [127]. DNA topoisomerases are crucial nuclear enzymes in DNA replication and repair. Many chemotherapeutic drugs target DNA topoisomerases and interfere with DNA replication to exert their anti-tumor activity [128]. It has been shown that GSTT1 expression is significantly upregulated in chemotherapy-resistant serous ovarian cancer (SOC) cells and that inhibition of GSTT1 expression negatively influenced the proliferation of SOC cells, thereby enhancing their sensitivity to paclitaxel/carboplatin [116]. Immunoprecipitation results showed a significant interaction between GSTT1 and Topo I in vitro, and these two enzymes expressed synergistically in drug-resistant cancer cells, suggesting that the mechanism of GSTT1-mediated drug resistance may be involved in DNA repair during chemotherapy of SOC cells [116]. To date, the mechanism of this interaction remains to be clarified.

#### 4.1.4. Effects of GSTs on Autophagy

Autophagy is a tightly regulated intracellular degradation process. As a dynamic circulator system, autophagy provides energy and components for cell renewal and maintenance of homeostasis [129]. The consequences of autophagy can be contradictory based on the stage of tumorigenesis. In non-tumor cells and at the early stage of tumor development, autophagy functions as a tumor suppressor, while in already-established tumors, autophagy promotes cancer cell survival [130]. However, the roles of autophagy in cancers vary with different types of tumors. In pancreatic cancer cells, the inhibition of autophagy leads to cell growth inhibition [131]. Fu et al. [113] showed that the chemoresistance to oxaliplatin in hepatocellular carcinoma cells might be mediated by GSTM1-regulated autophagy. In that study, GSTM1 silencing resulted in a significant decrease in the number of oxaliplatin-induced autophagic vesicles. Nevertheless, other studies have shown that activation of autophagy presented beneficial effects that facilitated lapatinib to overcome drug resistance and increase its toxicity in tumor cells [132]. Therefore, the different roles of autophagy and an in-depth understanding of the genetic backgrounds of specific tumor types are particularly important for the understanding of the involvement of GSTs in the autophagy process, which determines the fate of cancer cells.

#### 4.1.5. Effects of GSTs on Ferroptosis

Ferroptosis is a recently identified form of programmed cell death that is distinct from necrosis, apoptosis, and autophagy and was first described in 2012 [133]. It is considered to be an iron-dependent form of cell death and characterized by the involvement of lipid peroxidation, which ultimately leads to the rupture of the cytoplasmic membrane and the release of cellular contents. The role of ferroptosis in cancer treatment is gaining attention since it is recognized that induction of ferroptosis may be beneficial for more efficient elimination of cancer cells [134]. However, so far, ferroptosis-based therapy has been found to be effective in only a small number of cancer types, whereas most cancers encounter problems of ferroptosis resistance [135,136,137]. Wang et al. [138] first found that GSTZ1 could enhance sorafenib-induced ferroptosis by inhibiting the nuclear factor erythroid 2-related factor 2/glutathione peroxidase 4 (NRF2/GPX4) signaling pathway in hepatocellular carcinoma (HCC) cells. In this study, GSTZ1 was found to be downregulated in sorafenib-resistant HCC cells, while recovery of GSTZ1 enhanced sorafenib-induced ferroptosis in HCC cells. This suggests that GSTZ1 acts as a negative regulator of sorafenib resistance via the ferroptosis pathway. In contrast, microsomal glutathione S-transferase 1 (MGST1) was shown to negatively regulate and also promote resistance to ferroptosis in pancreatic ductal adenocarcinoma (PDAC) cells [139].

### 4.2. GSTs and Parkinson’s Disease

Parkinson’s disease is a movement disorder caused by degenerative changes in dopaminergic neurons in the substantia nigra of the skull, resulting in a decrease and deficiency of striatal dopamine. Oxidative stress has been reported to play a key role in the pathogenesis of PD [140]. Because of its relatively weak antioxidant capacity, the central nervous system is highly sensitive to oxidative stress, with the substantia nigra region being the most sensitive and vulnerable site [141]. A comparison of protein profiles using a quantitative proteomics technique revealed that GSTP1 is overexpressed in cortical neuronal cells in the late stages of PD [142]. In that study, GSTP1 overexpression was found to attenuate oxidative stress and ER stress as well as prevent rotenone-induced neurotoxicity. This suggests that GSTP1 may be able to delay disease progression in PD. Some studies suggested that the neuroprotective effects of GSTP1 may be related to its inhibition of JNK activation and prevention of the subsequent cell death cascade [23]. In addition, the overexpression of GSTS1 was shown to inhibit neurodegeneration [143]. Notably, GSTO1 may mediate the inflammatory response in the pathogenesis of PD and Alzheimer’s disease (AD) by participating in the regulation of interleukin-1β activity, and this inflammatory response is thought to be a contributing mechanism in the pathogenesis of PD and AD [144,145]. GSTM2 has been shown to be expressed in the substantia nigra of the human brain and exhibits a neuroprotective role by efficiently catalyzing the GSH-conjugation of ortho-quinone metabolite of dopamine, thereby protecting against its toxicity, redox cycling, and apoptosis, processes that have been associated with PD and schizophrenia [146]. The enzymatic activities of the GSTA, P, and T classes are substantially low or even negligible compared to GSTM2, while GSTM1 was slightly less effective than GSTM2.

### 4.3. GSTs and Epilepsy

Epilepsy is a chronic disease in which sudden abnormal discharges of neurons in the brain lead to transient brain dysfunction and muscular contractions. The pathogenesis of epilepsy is complex, and clinical data and experimental studies suggest that free radicals generated by oxidative reactions in mitochondria during disease onset may be the most critical cause of epilepsy pathogenesis. An association study showed that deficiency of GSTT1 is a risk factor for epilepsy, while genotypes of GSTM1 and GSTP1 showed no effect [147]. However, a very high GSTP1 expression was found in the neuroglia of epileptic foci in brain specimens from patients with refractory epilepsy when compared to patients with non-refractory epilepsy [148]. These GSTP1-positive astrocytes were widely present in the seizure lesions.

### 4.4. GSTs and Idiopathic Pulmonary Fibrosis

Idiopathic pulmonary fibrosis (IPF) is a chronic, progressive, fibrotic, and interstitial lung disease of unknown etiology, with the pathogenesis not fully elucidated. It has been shown that intracellular GST levels are increased in pulmonary fibrosis cells from IPF mice models and patients, suggesting that GSTs may play an important role in promoting pulmonary fibrosis formation [149]. The combination treatment with the GSTP inhibitor TLK117 and pirfenidone was found to be more effective than pirfenidone alone in a mouse model of pulmonary fibrosis. Pulmonary epithelial cell apoptosis promotes fibroblast activation and remodeling and may play a key role in the pathogenesis of IPF. McMillan et al. [150] demonstrated that S-glutathionylation of FAS by GSTP stimulates apoptosis of pulmonary epithelial cells, which may result in pulmonary fibrosis. These results showed that FAS-GSTP interaction was increased in lung epithelial cells of IPF patients and that the use of GSTP inhibitor TLK117 attenuated the level of S-glutathionylation and fibroblast remodeling [150]. This suggests that inhibition of GSTP in the airway may be a new strategy for the treatment of pulmonary fibrosis.

## 5. GST Inhibition in Disease Therapeutics

As introduced above, overexpression of GSTs in tumor tissues has been shown to increase tumor cell resistance to chemotherapies by multiple mechanisms. Additionally, GST upregulation has been suggested to play an important role in neurodegenerative diseases and pulmonary fibrosis. Therefore, targeting GST isozymes with specific inhibitors has been considered a potential therapeutic strategy for various diseases [150,151]. Over decades, GSTP, GSTA, GSTM, and GSTO inhibitors have been identified, and some of them have already been applied for clinical investigation or therapeutics. Representative examples and advances of research progress are described below.

### 5.1. GSTP Inhibitors

#### 5.1.1. Ethacrynic Acid and Its Derivatives

**Figure 6:**
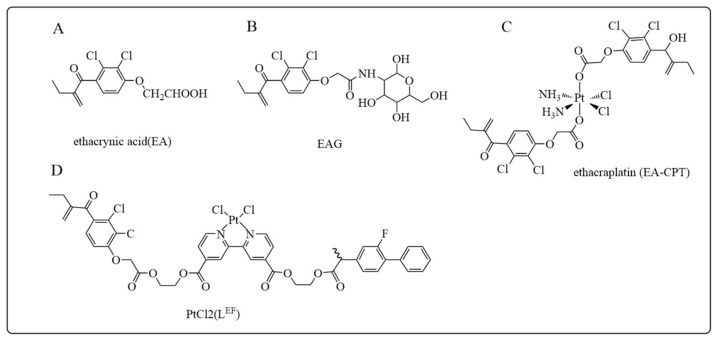
Structures of ethacrynic acid and its derivatives being developed as GSTP1 inhibitors: (**A**) Ethacrynic acid (EA); (**B**) EAG; (**C**) ethacraplatin (EA-CPT); (**D**) PtCl_2_ (LEF).

Ethacrynic acid (EA), Figure 6A, was first developed in 1963 as a potent diuretic for the treatment of patients with hypertension and intractable edema [152,153,154]. EA was found to be a potent inhibitor of GSTP, GSTA, and GSTM enzymes, with the most potent inhibition activity of GSTP1 [155]. The inhibitory effect of EA on GSTP1 is attributed to the α,β-unsaturated carbonyl group, which is capable of covalently binding to cysteine residues in the active site of GSTP1 following Michael addition reaction [7]. EA has been proven to exert anti-proliferative effects on tumor cells and increases the cytotoxicity of several alkylating agents such as melphalan, carmustine, mitomycin C, and nitrogen mustard. EA is both an inhibitor and substrate of GSTs. The glutathione conjugate of EA, EA-SG, exhibited a 10-fold higher inhibitory potency to GSTP1 than EA [156]. However, due to the lack of specificity and the strong diuretic side effects, the GST-targeted clinical usage of EA and its GSH conjugate is limited [154,157]. Punganuru et al. [157] designed and synthesized a non-diuretic EA analog, ethacrynic acid-glucosamine conjugate (EAG), Figure 6B, which targets tumor cells via the highly expressed glucose transporter 1. Cell survival assays showed that EAG was 3 to 4.5-fold more cytotoxic to human cancer cells when compared to EA. In response to GSTP1 overexpression-induced cisplatin resistance, a trans-Pt^IV^ carboxylate complex, ethacraplatin (EA-CPT), containing ethacrynate was developed (Figure 6C) [158]. By combining the advantages of cisplatin and EA, this compound efficiently alkylates the DNA of cancer cells and also inhibits GSTP1 and GSTA1 more effectively than EA [159]. In another study, the combination of ethacrynic acid and a flurbiprofen-like structure in the platinum complex PtCl2 (L^EF^) resulted in a high cancer cell selectivity and overcame the cisplatin resistance (Figure 6D) [160].

#### 5.1.2. NBDHEX and Its Analogues

**Figure 7:**
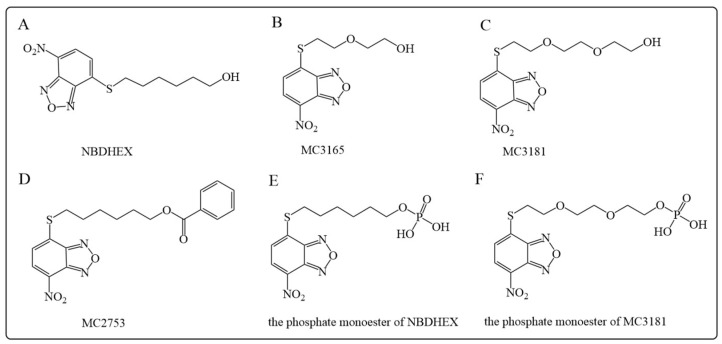
Structures of NBDHEX and its analogs as GSTP inhibitors: (**A**) NBDHEX; (**B**) MC3165; (**C**) MC3181; (**D**) MC2753; (**E**) the phosphate monoester of NBDHEX; (**F**) the phosphate monoester of MC3181.

6-(7-nitro-2,1,3-benzoxadiazol-4-ylthio)hexanol (NBDHEX) and its analogs are a class of non-glutathione (GSH) peptidomimetic compounds, Figure 7A. NBDHEX is a general inhibitor of GSTs, and the most potent inhibition of GSTP1. NBDHEX induces apoptosis in cancer cell lines alone or in combination with other antitumor agents, e.g., cisplatin, doxorubicin, vincristine, methotrexate, and temozolomide. The fact that NBDHEX is active in cell lines from various cancers, including leukemia, melanoma, osteosarcoma, and small-cell lung cancers, suggests it may be broadly applicable [161]. Several major mechanisms have been proposed for the cellular effects of NBDHEX. Firstly, NBDHEX induces apoptosis via activation of the JNK/c-Jun signaling pathway [162]. As a GSTP1 inhibitor, NBDHEX binds to the H site of GSTP1, forcing GSTP1 to be released from JNK protein, thereby inducing subsequent JNK phosphorylation, leading to tumor cell cycle arrest and apoptosis [163]. Secondly, NBDHEX has been found to dissociate the TRAF2-GSTP1 complex in human osteosarcoma cells (U-2OS), therefore inducing activation of JNK and p38 downstream signals and eventually apoptosis [11]. Therefore, NBDHEX was proposed as a potential treatment for cisplatin-resistant human osteosarcoma [155]. Thirdly, studies have shown that the compound can not only activate a variety of proapoptotic pathways but also acts as an inhibitor of autophagy in the late stage of melanoma, which may reduce tumor growth, metastasis, and progression [164]. Lastly, NBDHEX is not a substrate for the P-glycoprotein export pump but promotes cysteine-dependent apoptosis in cells overexpressing P-gp and may be used to treat P-gp-positive tumors [165].

In addition to GSTP1 inhibition, NBDHEX also showed a high affinity to GSTM2, which may lead to side effects [166]. Also, the poor water solubility limits its oral bioavailability. Therefore, researchers have developed several novel NBDHEX analogs to overcome the water solubility limitation and to increase the selectivity for GSTP1. Three NBDHEX analogs, MC3165, MC3181, and MC2753 (Figure 7B–D), are introduced as examples here [167,168]. It is shown that both MC3165 and MC3181 exhibited higher aqueous solubility, while MC3181 exhibited higher GSTP1 selectivity and higher cytotoxicity against osteosarcoma and melanoma cells [169,170]. MC2753 is a benzoate ester of NBDHEX; the water solubility and inhibition potency are similar to those of NBDHEX. The major advantage of MC2753 over NBDHEX is that the hydrophobicity of the side chain strongly affects the interaction between MC2753 and GSTP1 and does not require GSH to trigger the dissociation of the GSTP1-TRAF2 complex. Therefore, it may serve as a lead compound for the development of GSH-independent GSTP1 inhibitors [161]. Furthermore, recently, Di Paolo et al. [171] synthesized the phosphate monoesters of NBDHEX and MC3181 (Figure 7E,F), which showed high water solubility and potent GSTP1 inhibitory activity and therefore exhibited promising anti-proliferation effects against human melanoma and osteosarcoma cells, which is implicated as a potential clinical treatment for melanoma.

#### 5.1.3. TLK199 and TLK117

**Figure 8:**
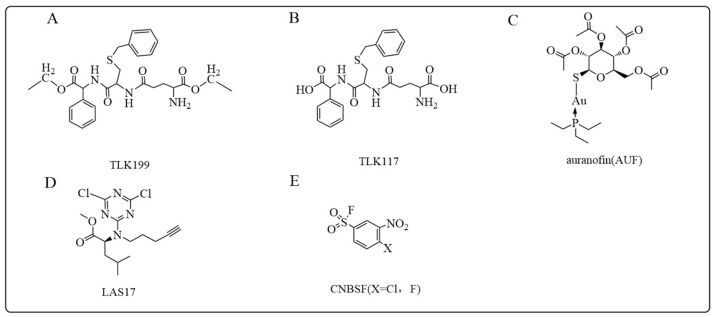
Structures of additional GSTP inhibitors introduced in this review: (**A**) TLK199; (**B**) TLK117; (**C**) auranofin (AUF); (**D**) LAS17; (**E**) CNBSF.

Ezatiostat hydrochloride (TLK199), a glutathione derivate and inhibitor of GSTP1, is developed for the treatment of myelodysplastic syndromes (MDS) (Figure 8A) [172]. TLK199 has been shown to stimulate the differentiation of primitive cells into mature monocytes, granulocytes, and erythrocytes and may prevent the generation of ineffective bone marrow in MDS [173]. As an ester prodrug, TLK199 undergoes hydrolysis reaction intracellularly to generate the active metabolite, TLK117. TLK117 selectively binds and inhibits GSTP1 with a Ki constant of 400 nM, substantially lower than the Ki range of 20 to 75 µM for GSTA and GSTM [174]. It promotes JNK phosphorylation of c-Jun and stimulates the proliferation of normal hematopoietic cells and/or apoptosis of malignant cells (Figure 8B) [22,175]. Moreover, TLK199 also acts as an inhibitor of MDR1 and enhances the effects of co-administrated anticancer drugs affected by efflux transport proteins [18,172]. Furthermore, TLK117-mediated GSTP1 inhibition may also block pulmonary fibrosis by inference of the JNK pathway [150].

#### 5.1.4. Auranofin

The antiarthritic gold(I) phosphine compound [(2,3,4,6-tetra-O-acetyl-1-(thio-κS)-β-D-glucopyranosato)(triethyl-phosphine)gold(I)], auranofin (AUF), was introduced clinically as an oral antiarthritic in 1979 (Figure 8C) [176]. There have been various studies demonstrating that auranofin and its analogs are promising anticancer agents for the treatment of cancers such as colorectal cancer and refractory sclerofibrosarcoma [177,178,179]. It has been reported that AUF exhibited an inhibitory effect on GSTP1 [180]. The inhibition potency of AUF on GSTP1 wild-type and cysteine mutants was similar, which suggested that unlike other inhibitors, GSTP1 inactivation by AUF is not related to cysteine residue binding. Further studies focusing on the mechanisms of the inhibitory effect of AUF on GSTs are needed. In addition, AUF is a strong inhibitor of the selenase thioredoxin reductase, which is associated with intracellular redox homeostasis and cytotoxic effects induced by oxidative stress [180].

#### 5.1.5. Arsenic Compounds

Trisenox (arsenic trioxide, As_2_O_3_) was initially approved for the treatment of acute promyelocytic leukemia (APL) in 2000, and following studies revealed that it is also effective against a variety of malignancies, including chronic lymphocytic leukemia, multiple myeloma, neuroblastoma, and gastric cancer [181,182,183,184,185]. However, GSTP1 overexpressing cancer cells are able to inactive As_2_O_3_ by catalyzing the formation of arsenic-GSH conjugates, which facilitate their elimination via MDR1 efflux in vitro, resulting in acquired resistance [186]. The organic derivative of arsenic, phenylarsenic oxide (PAO), was found to remain highly toxic in As_2_O_3_-resistant cell lines. PAO is described as one of the most potent GSTP1 inhibitors, with a K_i_ value of 90 nM, which binds both the enzyme active site and C101 at the dimer interface. The inhibition mechanisms of PAO were both GSH-dependent and independent. In the presence of GSH, the di-GSH-phenylarsenic complex binds to the G site and sequesters PAO at the dimer interface. In the absence of GSH, PAO binds to two cysteine residues at C47 and C101, respectively [187], and therefore blocks GSTP1 activity. This arsenical complex is considered a promising drug candidate for the treatment of APL, especially for patients with As_2_O_3_ resistance, and provides new insights for the development of GST inhibitors.

#### 5.1.6. LAS17

LAS17 is a dichlorotriazine-containing compound that can selectively and irreversibly inhibit GSTP1 in human cancer cells with high efficacy (Figure 8D). Unlike previously reported inhibitors targeting cysteine residues, the modification site of LAS17 in GSTP1 is a tyrosine residue (Y108) [188]. Louie et al. [70] found that LAS17 impaired cell survival of estrogen/progesterone/HER2 receptor-negative (triple negative) breast cancer cell lines. In addition, daily treatment of LAS17 slowed the growth of tumors implanted in immune-deficient mice. By analyzing the metabolomic changes induced by LAS17, it was demonstrated that GSTP1 greatly activates GAPDH-activity in the triple-negative breast cancer cells. The fact that activation of GAPDH by GSTP1 was independent of the presence of GSH or GSSG indicates that the activation resulted from protein-protein interaction, as was confirmed by a pulldown experiment. By inhibiting GSTP1, LAS17 treatment results in a decrease in GADPH activity, which plays an important role in glycolytic metabolism and oncogenic signaling pathways [73]. Therefore, the results with LAS17 illustrate that GSTP1 inhibition appears to be a novel therapeutic strategy to treat triple-negative breast cancer, which so far has a poor prognosis.

#### 5.1.7. CNBSF

1-Chloro-2,4-dinitrobenzene (CDNB) is a classic substrate of GSTs and is conjugated to GSH to form GS-DNB adducts. Shishido et al. [189] recently modified the structure of CDNB by replacing one of the nitro groups with sulfonyl fluoride (SF) to form chloronitrobenzenesulfonyl fluoride (CNBSF), a cell membrane permeable irreversible inhibitor of GSTP1 (Figure 8E). CNBSF first undergoes an aromatic substitution reaction with GSH, leading to the formation of a GSH adduct, which subsequently inactivates GSTP1 by forming a sulfonyl ester bond at Tyr108 [189]. CNBSF showed inhibitory effects on GSTP1 with IC_50_ values of 21 ± 1.3 µM (X = Cl) or 12 ± 0.4 µM (X = F). The design and synthesis of analogs of CNBSF are likely to contribute to the development of alternative G-site inhibitors of GSTs.

#### 5.1.8. Other GSTP Inhibitors

**Figure 9:**
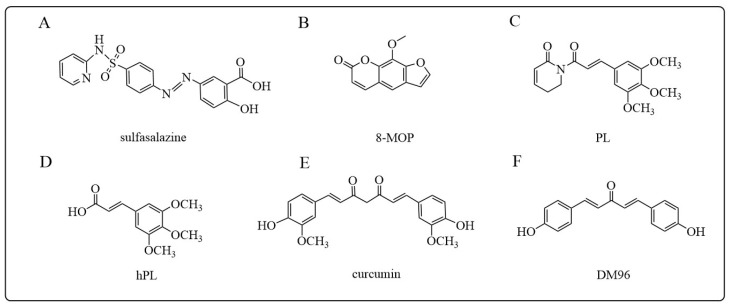
Structures of herbal components/originated GSTP inhibitors mentioned in this review: (**A**) sulfasalazine; (**B**) 8-MOP; (**C**) PL; (**D**) hPL; (**E**) curcumin; (**F**) the monosarbonyl curcumin derivative DM96 [198].

Sulfasalazine, an anti-inflammatory diseases reagent, can effectively inhibit GST isoenzymes A, M, and P, which was proposed to be a good candidate drug for regulating GSH/GST mediated drug resistances [190]. It was found that the combination of sulfasalazine with cisplatin improved the toxicity of cisplatin on human lung cancer cells overexpressing GSTP (Figure 9A) [191]. Besides the synthetic GSTP inhibitors as mentioned above, various ingredients originating from natural resources also showed inhibitory properties to GSTP. For example, 8-methoxypsoralen (8-MOP), originally extracted from the carrot family plant, is clinically used to treat skin diseases such as psoriasis and vitiligo. However, 8-MOP was found to act as a promising GST inhibitor (Figure 9B) [192,193]. It is shown that 8-MOP was able to bind tightly to the H site of GSTs by forming hydrophobic interactions with residues Phe-08, Tyr-108, Trp-38, Tyr-7, and Leu-52. Docking simulations showed that its interaction with the GSTP1 active site was more potent than NBDHEX. Piperlongumine (PL), derived from the amide of Capsicum annuum, is a biologically active alkaloid that showed potential anti-cancer activity (Figure 9C) [194]. PL is a prodrug, with hPL generated by intracellular hydrolysis being the active metabolite. hPL is able to conjugate with GSH to form a complex that tightly binds to the GSTP1 active site, thereby inhibiting cancer cell proliferation (Figure 9D) [195]. It has been shown that PL can selectively induce the death of head and neck cancer cells and increase cisplatin antitumor activity, involving the JNK and PARP death pathways [196]. Moreover, a computational simulation study predicted that curcumin is a potent competitive GST inhibitor and proposed a combination usage with chemotherapeutic agents for an improved efficacy [151]. Another study revealed that curcumin inhibits both mRNA and protein levels of GSTP1 and induces apoptosis by suppressing the GSTP1 transcriptional level (Figure 9E) [197]. Additionally, Pantiora et al. have found that the monosarbonyl curcumin derivative, DM96, is an effective GSTP1 inhibitor (IC_50_ = 5.45 ± 1.08 μM) (Figure 9E), with also showed potent cytotoxicity against prostate cancer cell line DU-145 (IC_50_ = 8.60 ± 1.07 μM), therefore was proposed as a decent chemical sensitizer to cancer cells [198].

### 5.2. GSTA Inhibitors

**Figure 10:**
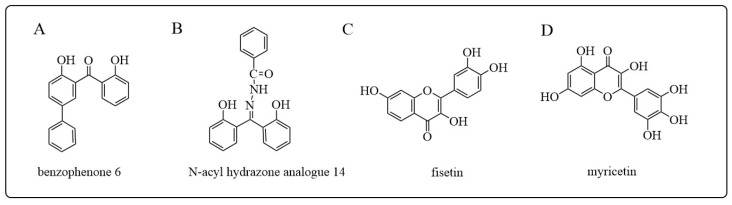
Structures of GSTA inhibitors mentioned in this review: (**A**) benzophenone 6 [199]; (**B**) N-acyl hydrazone analogue 14 [199]; (**C**) fisetin; (**D**) myricetin.

It has been shown that overexpressed GSTA1 can also be involved in the resistance of lung, ovarian, and stomach cancer cells to cisplatin. Therefore, selective inhibitors of GSTA are being developed, which may increase the sensitivity of these types of cisplatin-resistant tumors [112]. Perperopoulou et al. [199] reported that 2,2′-dihydroxybenzophenones and their carbonyl N-analogues are potential inhibitors of GSTA. Via the integration of GST inhibitor screening, enzyme inhibition kinetics, and molecular modeling approaches, they identified that 2,2′-dihydroxybenzophenones 6 and 8, as well as the N-acyl hydrazone analogues 14 and 16, exhibited satisfactory inhibitory potencies (IC_50(6)_ = 1.77 ± 0.10 μM; IC_50(8)_ = 0.24 ± 0.04 μM; IC_50(14)_ = 0.33 ± 0.05 μM; IC_50(16)_ = 0.18 μM). Examining the effects of these four compounds on the activity of human colon adenocarcinoma (Caco-2) cells, it was found that benzophenone 6 and N-acyl hydrazone analog 14 seem to be promising lead structures (LC_50(6)_ = 31.4 ± 0.4 μM; LC_50(14)_ = 87 ± 1.9 μM) (Figure 10A,B). Furthermore, different 2,2′-dihydroxybenzophenones and their carbonyl N-analogs showed distinct GSTA1 and GSTP1 isozymes inhibition specificity, with disubstituted benzophenones exhibited a minimal inhibition to GSTP1, whereas the inhibition potency to GSTA1 was still adequate [200]. In addition, natural flavonoids fisetin and myricetin are effective inhibitors of GSTA1, with IC_50_ values of 1.2 ± 0.1 µM and 2.1 ± 0.2 µM, respectively (Figure 10C,D) [201,202]. Fisetin not only inhibits the activity of GSTA1 in Caco-2 cells but also reduces the expression levels of GSTA1 mRNA and protein.

### 5.3. GSTM Inhibitors

**Figure 11:**
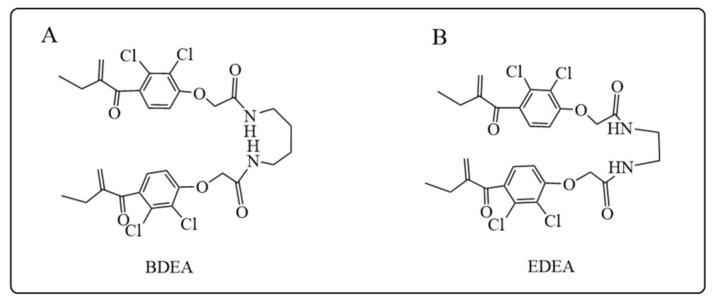
Structures of BDEA (**A**) and EDEA (**B**) as GSTM inhibitors.

Most GST inhibitors are monovalent inhibitors, with binding to either G- or H-site of the enzyme. This type of inhibitor normally exhibited high affinity, such as ethacrynic acid and its derivatives. In addition, bivalent inhibitors of GSTs are able to bind to both active sites and interact with both junction and cleft regions of the enzyme, thus showing even higher affinity and better isozyme selectivity compared to monovalent inhibitors [203]. A recent study linking ethacrynic acid with ethylenediamine and 1,4-butanediamine to obtain N, N′-ethyl-1,4-di-ethacrynic amide (EDEA) and N, N′-butyl-1,4-di-ethacrynic amide (BDEA), which are bivalent inhibitors of the membrane permeable glutathione S-transferase (Figure 11A,B) [204]. The bivalent GSH conjugates of BDEA and EDEA are able to produce inhibition by slow but tight binding to GSTM, which have the highest affinity for GSTM retrievable to date. Moreover, these two inhibitors were shown to be conventional inhibitors of GSTA and GSTP as well. The selectivity of BDEA toward GSTM was around 47-fold to that of GSTP and about 12-fold to that of GSTA, respectively. The affinity of EDEA for GSTM was about 15-fold higher than that of GSTP and GSTA. It is indicated that the two inhibitors might be promising probes for the biological and pharmacological roles of GSTM in cellular activity and as sensitizers for cisplatin-resistant ovarian cancer cells in chemotherapeutics, apart from the underlying mechanisms, are to be clarified. Nevertheless, drawbacks of EDEA and BDEA, such as high hydrophobicity and low solubility, as well as the strong binding to serum albumin, need to be solved in future structural optimization to obtain better membrane permeability and solubility [204].

### 5.4. GSTO Inhibitors

#### 5.4.1. Alpha-Tocopherol (Vitamin E)

**Figure 12:**
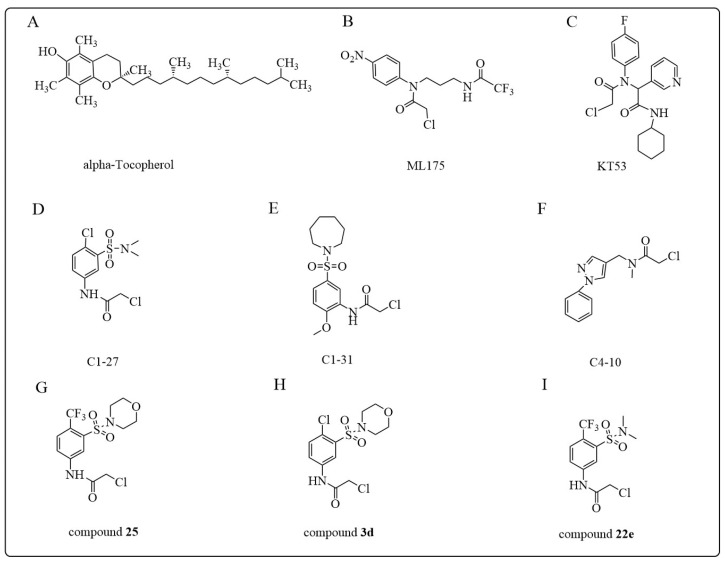
Structures of GSTO inhibitors: (**A**) alpha-Tocopherol; (**B**) ML175; (**C**) KT53; (**D**) C1-27; (**E**) C1-31; (**F**) C4-10; (**G**) compound **25** [214]; (**H**) compound **3d** [214]; (**I**) compound **22e** [214].

Vitamin E has been reported to prevent neurodegenerative diseases, and the association between vitamin E levels and brain health has been demonstrated, but the exact mechanism is unclear (Figure 12A) [205]. A previous study showed that interleukin 1β (IL-1β) is overexpressed in the brains of patients with Alzheimer’s disease (AD), while GSTO1 may be involved in the regulation of interleukin activity [144,206]. Therefore, therapeutic agents targeting GSTO1 and IL-1β might be capable of treating or ameliorating neurodegenerative diseases. Some researchers have suggested that the positive role of vitamin E in Alzheimer’s disease treatment is not caused by α-tocopherol alone but by another form of tocopherol or a combination of tocopherols [207]. In vitro studies showed α-tocopheryl phosphate and α-tocopheryl succinate (two forms of α-tocopherol supplementation) inhibited GSTO1 activity in a concentration-dependent manner [206,208]. The researchers speculated that the possible mechanism by which α-tocopherol ameliorates the progression of Alzheimer’s disease is the direct inhibition of GSTO1 and the subsequent inhibition of IL-1β activity. Further studies are needed to investigate the exact mechanisms of action of vitamin E in AD and the involvement of GSTO1 inhibition in this process.

#### 5.4.2. α-Chloroacetamide (CA)

As previously described, GSTO1 is overexpressed in human cancer cells and has been associated with chemotherapy resistance. The Scripps Research Institute Molecular Screening Center (SRIMSC) performed high throughput screening (HTS) of a variety of compounds by using fluorescence polarization–Activity-Based Protein Profiling (FluoPol-ABPP) and found that compounds containing the α-chloroacetamide fraction could act as selective GSTO1 inhibitors [209]. α-Chloroacetamide is a unique scaffold that can be used to target various proteins on cysteine residues. ML175 and KT53 are two potent and selective α-chloroacetamide GSTO1 inhibitors that are able to covalently modify the cysteine residue (Cys32) of GSTO1 (Figure 12B,C) [210,211]. Cancer cells treated with KT53 were shown to exhibit higher sensitivity to cisplatin in the study. Furthermore, Ramkumar et al. [212] identified three potent GSTO1 inhibitors containing chloroacetamide, namely C1-27, C1-31, and C4-10 (Figure 12D–F), via extensive small molecule screening, biochemical analysis, and X-ray crystallography. C1-31 and C4-10 interact with the H site by hydrophobic interactions, whereas C1-27 showed both hydrophilic and hydrophobic interactions with amino acid residues exhibiting the highest affinity. In addition, the authors predicted that these three compounds may also bind to GSTO2. These inhibitors could inhibit cancer cell growth, enhance the cytotoxicity of cisplatin, and act as a single drug to exert tumor growth inhibition in colon cancer models [212,213]. Recently, three more compounds (**3d**, **22e** and **25**) have been designed based on the C1-27 scaffold (Figure 12G–I) [210]. Compound **25** has the highest intracellular inhibitory activity with a *K*_inact_/*K*_I_ value of 2.3 × 10^4^ M^−1^S^−1^, while its elimination rate is slower than compounds C1-27, **3d**, and **22e**. Therefore, compound **25** is considered the most potent GSTO1-1 inhibitor reported up to date [214].

## 6. Conclusions and Perspectives

Over recent decades, the roles of GSTs in cancer occurrence, metastasis, and drug resistance have been studied extensively and intensively. Already-recognized mechanisms include the overexpression of GSTs in cancer cells mediated antineoplastics detoxification and MAPK signaling regulation. In fact, the modulation mechanisms of GSTs in cancer cells are complex and diverse, including the recently revealed functions in DNA repair, autophagy, and glycolysis. Interestingly, besides the high expression of GSTP1 in tumor tissues, its localization in tumor cells has also been associated with oncogenic effects. In various types of cancers, the high expression of GSTP1 in the cell nucleus is positively correlated with chemotherapy drug resistance and negatively correlated with the survival of tumor patients. A more comprehensive understanding of these mechanisms would reveal the core principles and significantly assist the development of new strategies for cancer therapeutics.

The selective inhibition of GSTP is probably the most well studied to date, where a large amount of GSTP inhibitors have been reported. One of the GSTP1 inhibitors with potential for clinical application is TLK199, which was renamed “*Ezatiostat*” after entering the clinical trial stage. Phase I and II clinical data show that ezatiostat has good tolerance and promotes hematopoietic activity in MDS patients. In addition, broad-spectrum GST inhibitors, such as 8-MOP and sulfasalazine, have been used clinically in combination with antineoplastic drugs for the treatment of chemotherapeutic-resistant cancers. Within this manuscript, we summarized the inhibitors developed for different GST isoenzymes, including some promising lead compounds such as CNBSF, benzophenone 6, and N-acylhydrazone analogue 14, and the potential applications in disease therapies. The design and development of these lead compounds will assist in the further optimization and finding of novel GST inhibitors with high potency and selectivity. Nevertheless, several significant limitations still hindered the development of the aforementioned compounds, e.g., low solubility, poor membrane permeability, poor specificity, and unclear in vivo efficacy and safety. Therefore, it is necessary to integrate the knowledge of medicinal chemistry, (bio)pharmaceutics, pharmacokinetics, pharmacodynamics, and toxicology for the continuous research and development of novel GST inhibitors for clinical applications.

## Figures and Tables

**Table 1:** Classification of cytosolic GST family members.

Class	Gene	Chromosome	Human Isoform	Tissue and Organ Distribution	References
α	*GSTA*	6p12.2	GSTA1-1	liver, kidney, adrenal gland, pancreas, testes, prostate	[14,15,16]
			GSTA2-2	liver, pancreas, kidney	[17,18]
			GSTA3-3	ovaries, testes, adrenal glands, placenta	[18]
			GSTA4-4	brain, placenta, skeletal muscle	[16]
			GSTA5-5	liver, kidney	[2]
μ	*GSTM*	1p13.3	GSTM1-1	liver, testes, brain	[16,18]
			GSTM2-2	brain, testes, heart	[16,18]
			GSTM3-3	testes, brain	[18]
			GSTM4-4	duodenum, intestine	[2]
			GSTM5-5	brain	[16]
π	*GSTP*	11q13	GSTP1-1	brain, heart, lungs, testes, pancreas, skin, kidney, bladder, prostate, colon	[16,19,20]
θ	*GSTT*	22q11.23	GSTT1-1	kidney, liver, small intestine, brain, lung	[2,16,18]
			GSTT2-2	liver	[18]
σ	*GSTS*	4q23.3	GSTS1-1	brain, heart, testicles	[16]
ω	*GSTO*	10q25.1	GSTO1-1	liver, heart,	[18]
			GSTO2-2	testicles	[21]
ζ	*GSTZ*	14q24.3	GSTZ1-1	liver, testicles	[16]

**Table 2:** Involvement of GSTs in multidrug resistance in cancer chemotherapeutics.

Isozyme Types	Types of Cancer	Anti-Tumor Drugs	References
GSTP1	Breast cancer, ovarian cancer, colorectal cancer, lung cancer, gastric cancer, glioma, human squamous cell carcinoma, glioblastoma multiforme (GBM), bladder cancer, osteosarcoma, mantle cell lymphoma (MCL), acute lymphoblastic leukemia (ALL), prostate cancer, esophageal cancer	Cisplatin, carboplatin, doxorubicin, cyclophosphamide, paclitaxel, docetaxel, melphalan, etoposide, oxaliplatin, fluorouracil, irinotecan, cytarabine, gemcitabine, bortezomib	[18,103,107,108,109,110]
GSTA1	Colorectal cancer, leukemia, lung cancer	Bacitracin, melphalan, chlorambucil, thiotepa, cyclophosphamide, imatinib, cisplatin	[18,104,106,111,112]
GSTM1	Intracranial tumors (ICT), liver cancer, melanoma	Thiotepa, oxaliplatin, vincristine	[18,97,107,113,114]
GSTM3	Breast cancer, glioblastoma multiforme (GBM)	BCNU, temozolomide (TMZ)	[18,103,105,115]
GSTO1	Breast cancer, pancreatic cancer, ovarian cancer	Cisplatin	[21,103]
GSTT1	Ovarian cancer, glioblastoma multiforme	Paclitaxel, carboplatin, BCNU	[18,116,117]
